# Chemokine Coreceptor Signaling in HIV-1 Infection and Pathogenesis

**DOI:** 10.1371/journal.ppat.1000520

**Published:** 2009-12-24

**Authors:** Yuntao Wu, Alyson Yoder

**Affiliations:** Department of Molecular and Microbiology, George Mason University, Manassas, Virginia, United States of America; The Scripps Research Institute, United States of America

## Abstract

Binding of the HIV-1 envelope to its chemokine coreceptors mediates two major biological events: membrane fusion and signaling transduction. The fusion process has been well studied, yet the role of chemokine coreceptor signaling in viral infection has remained elusive through the past decade. With the recent demonstration of the signaling requirement for HIV latent infection of resting CD4 T cells, the issue of coreceptor signaling needs to be thoroughly revisited. It is likely that virus-mediated signaling events may facilitate infection in various immunologic settings in vivo where cellular conditions need to be primed; in other words, HIV may exploit the chemokine signaling network shared among immune cells to gain access to downstream cellular components, which can then serve as effective tools to break cellular barriers. This virus-hijacked aberrant signaling process may in turn facilitate pathogenesis. In this review, we summarize past and present studies on HIV coreceptor signaling. We also discuss possible roles of coreceptor signaling in facilitating viral infection and pathogenesis.

## Introduction

Infection by the human immunodeficiency virus (HIV) causes severe depletion of the CD4 T cell population, which eventually leads to acquired immunodeficiency syndrome (AIDS). The selective nature of the infection immediately prompted speculation and the subsequent identification of the CD4 molecule as the main surface receptor for HIV entry [1],[2]. Nevertheless, it soon became apparent that CD4 alone did not seem to be sufficient to permit entry [3],[4]. A hunt for the elusive coreceptors ensued. In 1996, Berger's group first identified a G-protein-coupled receptor designated “fusin” as the elusive cofactor for HIV-1 entry [5]. “Fusin” was later renamed CXCR4 after the realization that its natural ligand is the CXC chemokine stromal cell derived factor 1 (SDF1) [6]. Before the identification of “fusin”, chemokines such as the CC chemokines RANTES, MIP-1α, and MIP-1β had been reported to suppress infection by the macrophage tropic (M-tropic) HIV-1 [7]. Thus, with the identification of CXCR4, it took little time to confirm that CCR5, the receptor for the aforementioned CC chemokines [8], was indeed also a coreceptor for the entry of M-tropic HIV-1 [9],[10],[11],[12],[13]. Following the discoveries of CXCR4 and CCR5, several other G-protein-coupled receptors have also been identified [14]. Nevertheless, the in vivo importance of these other coreceptors in viral infection and pathogenesis is less studied than that of CXCR4 and CCR5. This review focuses mainly on CXCR4 and CCR5 signaling.

## The Signaling Diversity of the Chemokine Coreceptors

The discovery of the HIV chemokine coreceptors opened up a new avenue for AIDS research. The fact that both CXCR4 and CCR5 are chemokine receptors raised interesting questions regarding the role of chemokine receptor signaling in viral infection and pathogenesis [5]. Early studies by Fauci's group demonstrated that the envelope from M-tropic but not T-tropic viruses can trigger calcium flux that was inhibited by pertussis toxin (PTX) or MIP-1β, which is suggestive of viral envelope-induced signaling transduction through CCR5 [15]. Subsequently, Davis et al. demonstrated that similar to SDF-1 and RANTES, both the T-tropic and M-tropic envelopes can induce rapid tyrosine phosphorylation of the protein tyrosine kinase Pyk2 through binding to CXCR4 or CCR5 [16]. Pyk2 phosphorylation is frequently associated with G protein signaling and calcium flux. These results provided early evidence that binding of the viral envelope to its chemokine corecptors, both CXCR4 and CCR5, not only mediates entry but also activates multiple intracellular signaling cascades, a process mimicking chemokine signaling through binding to their cognate receptors.

**Figure 1:**
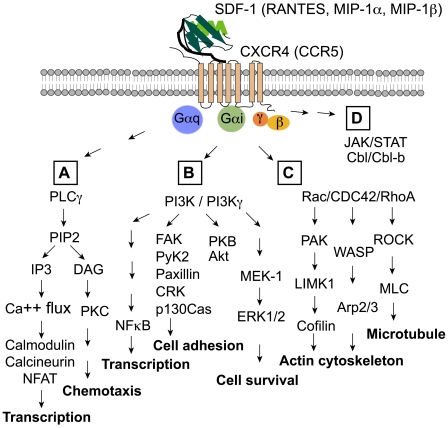
Chemokine receptor signaling pathways. SDF-1 binding to CXCR4 or RANTES/MIP-1α/MIP-1β binding to CCR5 activates G proteins (Gα particularly Gα_i_, Gα_q_, and Gβγ) and multiple downstream pathways. (A) Gα_q_ activates phospholipases such as phospholipase C-γ (PLC-γ), which hydrolyzes phosphatidylinositol-4,5-biphosphate (PIP2) to generate inositol triphosphate (IP3) and diacylglyerol (DAG), triggering calcium influx and the activation of kinases such as protein kinase C (PKC). (B) Gα_i_ activates phospholipases, phosphodiesterases, and the lipid kinase PI3K via Src-family kinases. Gβγ also activates PI3Kγ. PI3K activation stimulates downstream targets such as protein kinase B (PKB/Akt), NF-κB, mitogen/extracellular signal-regulated kinase (MEK-1), and extracellular signal-regulated kinase (ERK1/2). PI3K also triggers the tyrosine phosphorylation of focal adhesion complex components such as proline-rich tyrosine kinase (Pyk2), paxillin, Crk, and p130Cas. (C) GTP-bound Gβγ stimulates guanine nucleotide exchange factors (GEFs) such as TIAM1 and PREX1 specific for the Rho family GTPases (Rac/CDC42/RhoA). These GTPases activate pathways regulating cytoskeleton: Rac activates p21-activated kinase (PAK), which then activates LIM kinase (LIMK), leading to cofilin phosporylation and actin polymerization. CDC42 promotes actin assembly through the Wiskott-Aldrich Syndrome family protein (WASP) and actin-nucleating protein Arp2/3. RhoA activates Rho kinase (ROCK) , leading to myosin light-chain (MLC) phosporylation and microtubule rearrangement. (D) SDF-1 may also trigger Gα_i_-independent activation of the JAK-STAT pathways.

Chemokine receptor signaling is known to be diverse and is coupled to distinct signaling pathways that mediate cell migration, transcriptional activation, and cell growth and differentiation (Figure 1). For example, SDF-1 binding to the G-protein-coupled receptor CXCR4 activates heterotrimeric G-proteins (Gα and Gβγ). There are numerous classes of Gα (Gα_s_, Gα_i_, Gα_q_, Gα_12/13_), and CXCR4 seems to be specifically coupled to Gα_i_ and Gα_q_. Gα_q_ proteins activate phosphatidylinositol-specific phospholipases such as phospholipase C-γ (PLC-γ), which hydrolyzes phosphatidylinositol-4,5-biphosphate (PIP2) to generate inositol triphosphate (IP3) and diacylglyerol (DAG). These events lead to calcium flux and the activation of several PKC isoforms that have been shown to be important for SDF-1-induced chemotaxis [17],[18] (Figure 1A). Another pathway activated from SDF-1 binding to CXCR4 is through Gα_i_ protein activation, which inhibits adenylyl cyclases that in turn lead to a reduction in cAMP levels as well as the activation of phospholipases and phosphodiesterases. These events result in the activation of the lipid kinase PI3K via Gα_i_-coupled Src-family kinases as well as PI3Kγ through direct binding of Gβγ to the regulatory subunits of PI3Kγ [19],[20],[21]. Protein kinase B (PKB/Akt) and mitogen/extracellular signal-regulated kinase (MEK-1) and extracellular signal-regulated kinase (ERK1/2) are downstream of PI3K and function in cell survival and proliferation [21],[22],[23]. PI3K also stimulates the tyrosine phosphorylation of focal adhesion complex components such as proline-rich tyrosine kinase (Pyk2) [16], paxillin, and Crk [21],[24],[25], all of which are important for cell migration and cell adhesion. PI3K is also upstream of the critical nuclear transcription activator NF-κB, which regulates gene expression in response to inflammation and activates HIV proviral gene expression [21],[26] (Figure 1B).

SDF-1 binding to CXCR4 also results in GTP-bound Gβγ, which can stimulate guanine nucleotide exchange factors (GEFs) such as TIAM1 and PREX1 specific for Rho family GTPases such as Rac, Rho [27],[28],[29],[30], Cdc42 [31], and Ral [32]. These GTPases activate well-characterized downstream effector pathways, all of which modulate the cytoskeleton: Rac activates its downstream effector p21-activated kinase (PAK), which then activates LIM kinase (LIMK) [33], leading to the alteration of cofilin activity, which regulates actin turnover. Cdc42 activation promotes actin assembly through the Wiskott-Aldrich syndrome family protein's (WASP) activation of the actin nucleating protein Arp2/3 [34]. RhoA activation can lead to microtubule rearrangement through the Rho kinase (ROCK) activation of myosin light-chain phosphatase (MLCP) (Figure 1C). Finally, SDF-1 has been shown to activate perhaps some JAKs/STATs [35],[36],[37],[38], linking SDF-1-induced signaling to cytokine and growth factor–driven pathways regulating cell proliferation and differentiation. SDF-1 also activates Cbl/Cbl-b, which functions as an E3 ligase regulating cell signaling through ubiquitination [39]. In summary, most of these signaling molecules are components of the signaling transduction pathways mediating chemotactic responses for cytoskeleton rearrangement, cell polarization, and migration [19],[20],[30], as well as transcriptional activation, cell survival, and proliferation [21],[22],[40].

## The Signaling Diversity of the HIV-1 Envelope–Coreceptor Interaction

**Figure 2:**
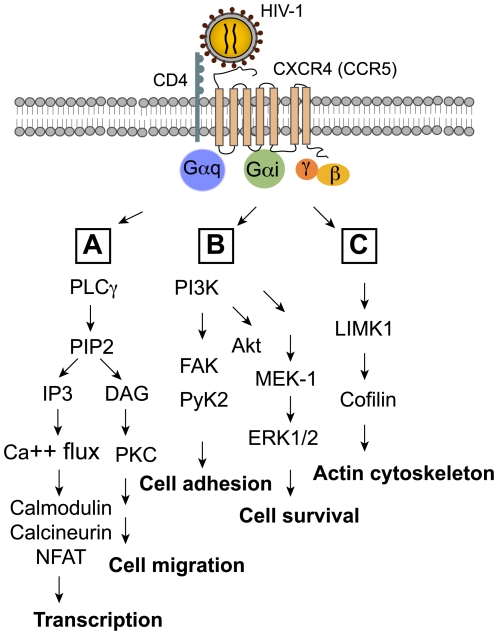
Components of the chemokine coreceptor signaling pathways activated by HIV-1 envelope. HIV-1 gp120 binding to CXCR4 or CCR5 activates a number of signaling molecules common to chemokine-mediated signaling pathways, including (A) PLC-γ-dependent calcium flux and NFAT nuclear translocation; (B) PI3K-dependent activation of FAK, PyK2, AKT, and ERK1/2; (C) the downstream targets of the Rho family GTPases such as LIMK1 and cofilin for actin rearrangement.

Consistent with the signaling diversity of chemokine–coreceptor interaction, binding of HIV-1 gp120 to CCR5 or CXCR4 has also been shown to trigger the activation of Pyk2 [16], PI3K, Akt [41],[42], Erk-1/2 [42], and CD4/CXCR4-dependent NFAT (nuclear factor of activated T cells) nuclear translocation [43] (Figure 2). Recently, gp120 was demonstrated to mediate chemotaxis, actin cytoskeleton rearrangement [42],[44], and the activation of an actin depolymerization factor, cofilin, to increase the cortical actin dynamics in resting CD4 T cells [44].

Given this array of targets that can be activated by gp120 in vitro, one question is whether these signaling events are physiological at the low gp120 dosages present in vivo. The presumption based on plasma viral load is that the physiological levels of gp120 are likely to be significantly lower than most of those used in in vitro experiments [45]. However, the in vivo concentrations of gp120, particularly the local gp120 concentrations in tissues, are difficult to measure, and a large percentage of the virus in the body is present in lymphoid tissues. Therefore, the compartmentalized viral concentrations could be very high regardless of the plasma viral loads [46]. Thus, the judgment of whether or not an experimental gp120 dosage is physiological is largely arbitrary at best. On the other hand, a recent experiment suggested that a few HIV particles might be sufficient to trigger signaling through CXCR4 [47].

In our recent studies of gp120 signaling in peripheral resting CD4 T cells, we did notice dosage- and conformation-dependent differences in gp120 signaling [44],[48]. At high gp120 dosages, gp120 acts more like SDF-1, triggering rapid cofilin phosphorylation and actin polymerization that was followed by cofilin dephosphorylation and actin depolymerization [42],[44]. At lower dosages, gp120 was incapable of triggering such rapid changes, but was able to mediate gradual cofilin dephosphorylation and actin depolymerization [44],[48]. These differences may play different roles in various settings during the course of HIV infection.

## Roles of HIV-1 Envelope–Coreceptor Signaling in HIV Infection

The direct involvement of chemokine coreceptor signaling in HIV infection has been speculated right from the identification of the chemokine coreceptors [5]. Within a year of the initial discovery, multiple groups started to test the requirement of coreceptor signaling in HIV entry and subsequent replication steps. Cocchi and colleagues were the first to demonstrate that inhibition of coreceptor signaling through PTX neither inhibited HIV-1 replication nor the ability of chemokines such as RANTES to block HIV-1 entry into PM1 cells [49]. Similarly, Farzan et al. further created three CCR5 mutants that abolished its signaling ability to mobilize calcium but detected minimal effects on viral entry and replication in Hela-CD4 cells [50]. These results were corroborated by several other groups using cell lines transfected with either CCR5- or CXCR4-signaling-defective mutants [51],[52],[53],[54],[55]; the results largely suggested that the signaling function of the chemokine coreceptors is an independent function not required for viral entry or replication. Nevertheless, in the following decade, a significant number of studies have been published describing modulation of cellular functions by HIV gp120 signaling, ranging from causing neurotoxicity to promoting apoptosis. Therefore, the relevance of chemokine coreceptor signaling to viral infection itself remained an open question. With this significant issue unresolved, there were also sporadic findings implying that chemokine coreceptor signaling might be important for viral replication. For example, prestimulation of macrophages or CD4 T cells with CC-chemokines was found to enhance HIV replication [56],[57]. Similar stimulation with viral gp120 can even induce viral replication in cultures of resting CD4 T cells of infected patients [58]. These positive effects of coreceptor signaling were also reflected in facilitating HIV infection of non-natural targets; the replication of some HIV-1 isolates in macaque cells was blocked at a step after entry and reverse transcription but prior to the nuclear import of the preintegration complex, and this block could be relieved by the expression of human coreceptor CCR5 or CXCR4 in macaque cells [59]. These results suggested a possible involvement of coreceptor signaling in a post entry step. In agreement with this hypothesis, Mori and colleagues demonstrated that three to nine amino acid changes in the envelope of SIVmac293 conferred upon the virus the ability to replicate 100–1,000 times more efficiently in macrophages. Importantly, the amino acid changes in the envelope did not enhance virus entry, but rather affected some post entry steps [60]. Furthermore, Arthos and colleagues [61] demonstrated that there was a direct correlation between the capacity of viral envelopes to initiate signaling and the ability of the same viruses to infect cells. They also demonstrated that a signaling-deficient R5 HIV-1, 92MW959, entered macrophages but failed to replicate, and the block was at a post entry step. In parallel, the density of CCR5 on the cell surface also appeared to correlate with the capacity of the coreceptor to transduce signals, and this capacity directly impacted viral post entry processes such as reverse transcription and integration [62]. Recently, Grainger and Lever [63] used a family of new chemokine inhibitors to block chemokine receptor signaling without affecting receptor binding. When tested in HIV-infected cells, these inhibitors inhibit HIV-1 infection without blocking gp120/CCR5 interaction [63], and presumably, these inhibitors also inhibited viral replication at steps post binding and entry.

Despite growing evidence supporting a possible role for coreceptor signaling, contradictory findings remained. Amara et al. compared R5 HIV infection of CCR5 wild-type and signaling-deficient T cells derived from CCR5Δ32 individuals [55]. In this study, signaling-deficient CCR5 supported R5 HIV replication to an extent similar to the wild-type CCR5, suggesting again that G protein signaling through CCR5 plays no role in R5 HIV-1 replication. An important aspect of this study was the use of PHA to activate primary T cells for transduction. In contrast, Lin et al. used unstimulated primary T cells in a similar experiment and showed that the signaling-deficient CCR5 T cells were impaired in their support of HIV-1 infection [64]. Importantly, in order to observe the inhibition, cells in this experiment had to be rested for ten days after transduction before HIV-1 infection. These data are consistent with an earlier observation that the B-oligomer of PTX, although not directly inhibiting coreceptor signaling thourgh Gαi, diminishes the R5 virus-mediated receptor capping and reverse transcription [65]. These inhibitory effects can only be observed in primary monocyte-depleted PBMCs but not in transformed PM1 cells [65]. It is likely that much of the seemingly contradictory observations in different studies may arise from the use of cells in different activation states. When studies used activated T cells or transformed cell lines, they largely observed that HIV-1 envelope–coreceptor signaling was dispensable. Since a majority of T cells and macrophages in vivo are in resting and noncycling states, it is critical that the study of HIV-initiated signaling is investigated using similar cells.

**Figure 3:**
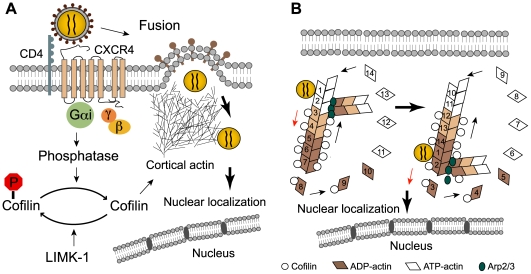
HIV-1 envelope-CXCR4 signaling triggers cofilin activation to promote cortical actin dynamics and HIV nuclear migration. (A) HIV-1 envelope binding to CD4 and the chemokine coreceptor, CXCR4, triggers membrane fusion and signaling transduction. The initial viral contact with CD4 and then CXCR4 may trigger rapid actin polymerization to facilitate CD4/CXCR4 cocapping for fusion and entry. Following fusion, the viral preintegration complex (PIC) may be directly anchored onto F-actin to facilitate reverse transcription. Subsequent actin activity mediated by cofilin activation through CXCR4 promotes viral nuclear migration. (B) Model of HIV PIC migration along the cortical actin filaments. It is possible that cofilin activation increases actin treadmilling, which promotes the movement of the viral PIC across the cortical actin barrier, allowing PIC to gain access to the perinuclear or nuclear region. The number is arbitrarily assigned to an actin monomer to demonstrate the actin movement during treadmilling. (Figure 3 is modified from [44] with permission).

Recently, we examined the requirement for CXCR4 signaling in HIV-1 latent infection of human resting CD4 T cells using an in vitro system to mimic in vivo latent infection of resting T cells [44]. Using this system, we observed an absolute requirement of CXCR4 signaling for HIV-1 latent infection of resting CD4 T cells. This requirement was even maintained in resting T cells exposed to certain cytokines such as IL-2 and IL-7, suggesting that CXCR4 signaling could potentiate infectivity even in certain cytokine-enriched microenvironments, such as lymphoid tissues. We also identified the molecular mechanism of this signaling requirement, and demonstrated that the static cortical actin in noncycling, resting CD4 T cells represents a unique barrier for viral post entry migration. To overcome this restriction, HIV-1 relies on viral envelope and the Gαi-dependent signaling from the chemokine coreceptor CXCR4 to activate a cellular actin-depolymerizing factor, cofilin, to increase the cortical actin dynamics (Figure 3). This unique requirement for coreceptor signaling can only be observed in noncycling, resting CD4 T cells because in transformed or activated T cells cofilin is constitutively active to facilitate the cell cycle–driven actin remodeling that renders CXCR4 signaling unnecessary [44].

With the identification of the molecular mechanism of CXCR4 signaling, the remaining issues are to confirm the requirement for CCR5 signaling, and to determine the molecular basis for this requirement. There appears to be a clear distinction in the requirement for signaling between R5 and X4 viruses. For example, the B-oligomer of PTX inhibits R5 virus at entry and reverse transcription, but inhibits the X4 virus at multiple steps post reverse transcription [65],[66]. The R5 virus predominates at the early time of HIV infection, largely infecting macrophages and active memory CD4 T cells [67] in the gastrointestinal (GI) tract and lymph nodes [68]. Both types of cells support productive viral replication and do not have the restrictions usually seen in resting CD4 T cells. It is unknown whether HIV-mediated cofilin activation through CCR5 may also occur in active memory T cells and macrophages, since cells that are actively cycling and migrating normally disassemble actin cytoskeleton themselves, leaving the cells naturally susceptible to HIV-1 infection. If required, the CCR5 signaling might be involved in several early processes: initial fusion and entry, uncoating, viral DNA synthesis, or subsequent viral gene transcription. Chemokine receptor signaling also triggers rapid actin polymerization [42],[44], which may be important for the cocapping of CD4 with CCR5 or CXCR4 [69] and for the efficient synthesis of viral DNA [44],[70]. This early actin polymerization appears to be involved in assembling high concentrations of CD4 and CCR5 or CXCR4 at the plasma membrane, which facilitate gp120 binding and viral entry (for a review, please see [71]). Nevertheless, it is unlikely that this immediate actin activity is triggered through CCR5 or CXCR4 exclusively. Rather, it may be mediated through the initial gp120 contact with CD4 and then facilitated through further gp120 contact with CCR5 or CXCR4 [72] (Figure 3). Also, sufficient actin contact with the core following fusion is probably critical for rapid uncoating, or a subsequent particle conformational change that is necessary for optimal reverse transcription. Indeed, excessive actin depolymerization inhibits viral replication, and artificially increasing the cortical actin density through small interfering RNA (siRNA)-mediated cofilin knockdown enhances viral DNA synthesis [44]. In transformed cell lines, the requirement for CCR5 signaling in viral replication was not observed, likely because the cortical actin itself is dynamic and minimally affected by inhibiting CCR5 signaling [44]. In addition, the presence of CD4 signaling may also compensate some of the early requirements for actin activity. Interestingly, when the density of CCR5 was artificially increased on transformed HOS-CD4 cells, a direct correlation between CCR5 density and viral DNA synthesis was observed, and this correlation was dependent on PTX-sensitive Gαi signaling [62].

CCR5 signaling has also been known to trigger distinctive signaling cascades that activate kinases and transcription factors associated with cell activation [43],[73],[74]. For example, R5 envelopes can induce the expression of genes belonging to MAPK signal transduction pathways and genes regulating the cell cycle [74]. R5 envelopes can also activate NFATs and induce their translocation into the nucleus [43]. Because the HIV long terminal repeat (LTR) encodes NFAT recognition sites, NFAT activation likely enhances viral transcription directly from the LTR promoter, especially at early time points when cellular conditions are limiting. For example, the microenvironment in the GI tract where the R5 viruses infect active memory T cells is filled with inhibitory cytokines such as TGF-β [75], which can reorganize cytoskeletal structure [76] and inhibit the activation of major transcription factors such as NFκB [77]. The contribution of CCR5 signaling to viral transcription could probably be observed in suboptimally activated cells, but may not be seen in highly active transformed cell lines.

## Possible Roles of HIV-1 Envelope–Receptor Signaling in Viral Pathogenesis

It has long been suggested that HIV-1 envelope plays a central role in HIV-mediated CD4 T cell depletion and pathogenesis. Prior to the identification of the chemokine coreceptors, gp120 was proposed to directly trigger activation-dependent T cell apoptosis through its binding to surface CD4 [78]. Later, when the chemokine coreceptors were discovered, gp120 binding to CCR5 or CXCR4 was also proposed to trigger the apoptosis of CD4 cells, CD8 T cells [79],[80], and neurons [81]. There was considerable complexity and discrepancy regarding possible mechanisms of gp120-mediated apoptosis in T cells, in terms of possible involvements of Gαi-dependent pathways and caspases [80],[82], as well as the involvement of autophagy [83]. Envelope-primed apoptosis has been implicated as directly contributing to the depletion of either infected or uninfected CD4 T cells during disease progression [78],[84],[85]. Nevertheless, latently infected resting T cells were frequently detected persisting in patients [86], and the half life of these cells can be as long as 3 to 4 years [87]. These in vivo findings suggested that although gp120 can trigger T cell apoptosis in certain circumstances, there must be mechanisms that may prevent this process from occurring during the initial virus–cell engagement [88].

HIV envelope–chemokine receptor signaling has been shown to mediate chemotaxis in both CD4 and CD8 T cells [15],[42],[44],[89],[90], a property directly related to the role of chemokine receptor signaling. The viral envelope has also been suggested to do the opposite by repelling T cells [89], a process resembling chemofugetaxis, in which high concentrations of chemokines such as SDF-1 drive away T cells [91]. It has been proposed that this capacity of gp120 repels antigen-specific cytotoxic T lymphocytes from viral infection sites to evade immune effector mechanisms [89]. Similar functions of gp120 in modulating the immune system have also been suggested. For example, persistent gp120 stimulation induces CXCR4-dependent T cell anergy [92], as well as CCR5- and CXCR4-dependent macrophage activation and the secretion of proinflammatory cytokines [93]. Many of these gp120-induced phenotypes resemble the pathogenic features of HIV-induced T cell dysfunction and chronic immune activation [92],[94],[95],[96],[97]. However, the molecular targets and signaling mechanisms directly responsible for these phenotypes remain largely uncharacterized. Recently, we identified cofilin as one of the primary downstream targets of gp120–CXCR4 signaling in human resting CD4 T cells [44]. We also found that in the resting CD4 T cells of infected patients, cofilin activity is aberrantly upregulated [48]. HIV-mediated cofilin activation likely results from a bystander effect since a majority of resting CD4 T cells in the peripheral blood of infected patients contain no virus (0.2–16.4 cells latently infected with HIV per 10^6^ resting CD4 T cells [98]). Although not directly infected, these residual CD4 T cells in patients are also known to carry numerous functional abnormalities such as loss of T helper function [94], anergy [92],[95], increased T cell proliferation [99], and abnormal T cell homing and migration [89],[100].

In the human immune system, cofilin is involved in two hallmark activities of T cells, namely chemotaxis and T cell activation [101]. In chemotaxis, cofilin is the driving force for promoting the cortical actin dynamics central to cell migration [33]. In antigen-specific T cell activation, cofilin is activated through CD2/CD28 co-stimulation, and plays a critical role in actin reorganization and the formation of the immunological synapse that is required for sustaining T cell activation [102]. It is possible that cofilin dysregulation could result in CD4 T cell abnormities that may contribute to T cell depletion and immune deficiencies. Severe effects of altered actin dynamics on the human immune system have been well documented. For instance, a genetic defect of WASP that affects actin dynamics causes immunodeficiency [103]. HIV-mediated aberrant activation of cofilin strikingly resembles the activation of cofilin detected in migratory T lymphoma cells [104]. This resemblance may indicate a similar abnormal migratory behavior that could result in the eventual destruction of peripheral CD4 T cells in lymph nodes or tissues of HIV patients.

The utilization of different chemokine coreceptors, either CCR5 or CXCR4, for entry largely differentiates HIV into two distinctive phenotypes with either M- or T-tropism. The high pathogenic potential of the late emerging T-tropic viruses in causing rapid CD4 depletion is clearly a demonstration of the pathogenic significance of the CXCR4-engaging viruses [105],[106],[107]. Nevertheless, a remaining question for the importance of CXCR4 signaling in HIV pathogenesis is the lack of X4 viruses in some patients. There are only approximately 50% patients who experience the conversion from R5 to X4 viruses at later stages of disease. It is possible that in these patients, a complete viral switch from M- to T-tropism may never occur, but constant viral mutation may generate intermediate viruses that can engage and signal through CXCR4. It has been known that one or two amino acid changes in the V3 loop of the viral envelope may confer CXCR4 binding [108], although successful fusion and entry often require more than two mutations or mutations even outside of the V3 loop [109]. It is also possible that these hypothetical X4-signaling viruses may trigger signaling transduction and cause CD4 T cell dysfunction without actually replicating in them (for further reading on this hypothesis, please see [110]). In comparison with the highly pathogenic T-tropic viruses, these M-tropic, X4-signaling viruses may cause slower CD4 depletion and disease progression.

## Conclusions and Perspectives

Understanding HIV envelope–coreceptor interaction holds the key to unlocking the mystery of HIV-mediated CD4 depletion and pathogenesis. The signaling capacity of the viral envelope, along with its pathogenic potential, has been a subject of great interest and intense investigation. Yet, the critical role of the coreceptor signaling in HIV disease had not been clearly defined. This lack of appreciation has been largely attributed to the complexity of the chemokine signaling network itself, as well as the complication of many in vitro systems used, in which cellular states are derailed from the genuine signaling circuitries of natural viral targets in the body. Given these complexities, a critical function of the coreceptor signaling in promoting viral infection has only recently been demonstrated in the latent infection of human resting CD4 T cells. The deciphering of the molecular mechanisms involved starts to offer exciting fresh perspectives and opens a new avenue for examining HIV pathogenesis. However, a great deal is yet to be tested regarding the in vivo importance of coreceptor signaling in mediating CD4 T cell dysfunction and pathogenesis.

## Gene Accession Numbers

Human CXCR4 – Entrez Gene ID # 7852; Human CCR5 – Entrez Gene ID # 1234; Human non-muscle Cofilin 1 – Entrez Gene ID # 1072.
